# Dynamic Modeling of a Proton-Exchange Membrane Fuel Cell Using a Gaussian Approach

**DOI:** 10.3390/membranes11120953

**Published:** 2021-12-01

**Authors:** Catalina González-Castaño, Leandro L. Lorente-Leyva, Janeth Alpala, Javier Revelo-Fuelagán, Diego H. Peluffo-Ordóñez, Carlos Restrepo

**Affiliations:** 1 Department of Engineering Sciences, Universidad Andres Bello, Santiago 7500971, Chile; inv.cet@unab.cl; 2 Postgraduate Center, Universidad Politécnica Estatal del Carchi, Tulcán 040101, Ecuador; leandro.lorente@upec.edu.ec; 3 Artificial Intelligence for Electrical Engineering Research Program, SDAS Research Group, Ben Guerir 47963, Morocco; janeth.alpala@sdas-group.com; 4 Department of Electronics Engineering, Faculty of Engineering, Universidad de Nariño, Pasto 52001, Nariño, Colombia; javierrevelof@udenar.edu.co; 5 Modeling, Simulation and Data Analysis (MSDA) Research Program, Mohammed VI Polytechnic University, Ben Guerir 47963, Morocco; peluffo.diego@um6p.ma or; 6 Faculty of Engineering, Corporación Universitaria Autónoma de Nariño, Pasto 52001, Nariño, Colombia; 7 Department of Electromechanics and Energy Conversion, Universidad de Talca, Curicó 3340000, Chile

**Keywords:** Gaussian model, proton exchange membrane fuel cell, diffusive model, evolution strategy, voltage-current dynamic response

## Abstract

This paper proposes a Gaussian approach for the proton-exchange membrane fuel cell (PEMFC) model that estimates its voltage behavior from the operating current value. A multi-parametric Gaussian model and an unconstrained optimization formulation based on a conventional non-linear least squares optimizer is mainly considered. The model is tested using experimental data from the Ballard Nexa 1.2 kW fuel cell (FC). This methodology offers a promising approach for static and current-voltage, characteristic of the three regions of operation. A statistical study is developed to evaluate the effectiveness and superiority of the proposed FC Gaussian model compared with the Diffusive Global model and the Evolution Strategy. In addition, an approximation to the exponential function for a Gaussian model simplification can be used in systems that require real-time emulators or complex long-time simulations.

## 1. Introduction

During recent years, fuel cells (FC) have been one of the most researched topics due to several characteristics suitable for large-scale energy storage. Compared with other technologies, such as wind and photovoltaic (PV) generation, fuel cell technology does not have geographic requirements [1]. Therefore, this technology is rapidly expanding, and several lines of research have emerged associated with different sectors. The main sectors behind the development of fuel cells are: transportation, residential heat production, commerce and industry, electric power, and renewable energy [2,3,4,5]. A fuel cell system consists of generating electric power from the chemical reaction between hydrogen and oxygen or natural air in catalyst cells [6,7]. The proton-exchange membrane fuel cell (PEMFC) has received significant attention from researchers. It is considered one of the best FC alternatives for applications in different sectors due to its relatively low-temperature operation, which assures fast start up, the highest efficiency and zero pollution emission [8,9]. Due to the numerous applications of PEMFC, an accurate model is necessary for understanding the dynamic process and the behavior of this fuel cell [10].

For different applications, an FC simulation or hardware emulation is necessary for prototype design, testing, and fault diagnosis, reducing the overall cost [11]. Existing FC simulators and emulators are based on curve fitting, cell equivalent-impedance model, and employment of artificial intelligence algorithms using a massive amount of data from a real FC battery [12,13,14,15]. Therefore, several models that describe the phenomena occurring within fuel cells have been developed [16,17].

The FC models comparison, based on analytical and numerical procedures, is shown in Table 1. The models presented in [7,16,18,19,20,21,22] consider a linear steady-state response, ignoring the voltage-current dynamic response. Therefore, these models are not viable for the analysis of the dynamic FC process. On the other hand, most of the models contemplate an analytical approach based on the physical system of the FC. These approaches need several variables to evaluate the model, such as the operating absolute temperature of the fuel cell ($f_{fc}$), the operating current of the fuel cell ($i_{fc}$), the partial pressures of hydrogen and oxygen at the input channels of the fuel cell stack ($P_{H2}$ and $P_{O2}$), and the resistance of membrane surface ($R_{m}$). However, due to implementation expenses, using multiple variables for its evaluation increases the development costs and the requirements of the high-processing device. As an alternative method for dynamic modeling, this work introduces a novel FC model based on a Gaussian approach. Such an approach consists of a multi-parametric Gaussian model solved by an unconstrained optimization formulation [23]. Specifically, the optimal solution comes from a trust-region-based non-linear least squares optimizer. The model includes both the steady-state and transient responses of the actual fuel cell. Moreover, the model only needs the operating current of the fuel cell to predict the output voltage behavior; this is true for large-signal step-type variations at any point of the whole operation range of the current. Thus, the model is suitable to be implemented in a low-cost (digital signal controller) DSC. Based on this state-of-the-art review, the following are the main contributions of this paper:

- Provides a novel FC model to estimate the output voltage behavior from the operating current of a fuel cell for steady-state and dynamic responses.
- The training complexity of the algorithm is medium, which makes it easily adaptable to different profiles for testing.
- The proposed FC model can be used in computer simulations and hardware emulators due to its simple implementation using an approximation to the exponential function.
- A commercial Nexa Fuel Cell Power Module is used to validate the proposed FC model.
- The results are compared using analytical and numerical techniques under the same data acquisition parameter to ensure a fair comparison between the models.
- The obtained results prove the effectiveness of the proposed FC model compared with the Evolution strategy [8] and the diffusive model [24].

This paper is structured as follows: Section 2 outlines the multi-parametric Gaussian model considered in this work. Next, Section 3 describes the unconstrained non-linear optimization formulation that determines the optimal solution for the model parameters. Afterward, Section 4 gathers the experimental results and draws the discussion. Finally, the conclusions are presented in Section 5.

## 2. Gaussian Model

The Gaussian model–better known as Gaussian peaks–is widely used in different areas of science and applied research, such as biology, physics, chemistry, and engineering, when curve fitting is required [29,30].

**Figure 1:**
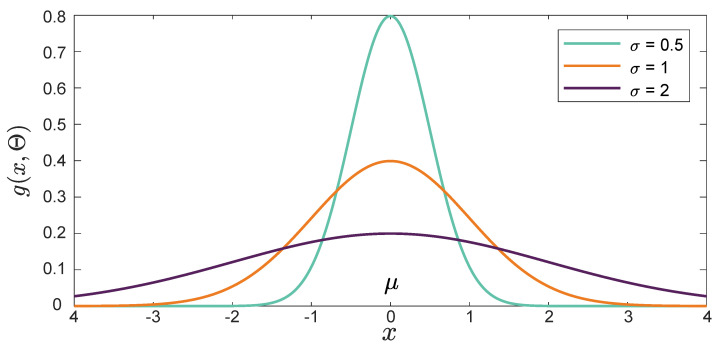
Resulting curve of the Gaussian peaks method when varying the values of the free parameter σ.

Let g(x,Θ) be the Gaussian model in one dimension, and Θ=[A,μ,σ] the vector of parameters to be estimated, which can be expressed using Equation (1)

(1) $$\begin{array}{c} g\left(x,\Theta \right)=Ae^{-{\left(\left(x-\mu \right)/\sigma \right)}^{2}}, \end{array}$$

where *A* is the amplitude of the curve, μ represents the position of the center of the peak, and σ is a free parameter controlling the width of the curve. Figure 1 illustrates the effect of the Gaussian peaks method at different values of σ.

In general, a multi-Gaussian model, denoted as $f_{\mathrm{Gauss}}\left(x\right)$, is described by the following Gaussian series:

(2) $$\begin{array}{cc} f_{\mathrm{Gauss}}\left(x\right) & =\sum_{i=1}^{N}g_{i}\left(x,\Theta_{i}\right),\\ \; & =\sum_{i=1}^{N}A_{i}e^{-{\left(\left(x-\mu_{i}\right)/\sigma_{i}\right)}^{2}}\\ \; & =A_{1}e^{-{\left(\left(x-\mu_{1}\right)/\sigma_{1}\right)}^{2}}+\\ \; & \;\;\;\;\;\vdots \\ \; & \;+A_{N}e^{-{\left(\left(x-\mu_{N}\right)/\sigma_{N}\right)}^{2}}. \end{array}$$

where *N* is the considered number of peaks.

## 3. Unconstrained Nonlinear Optimization

Given a set of data points $\left(x_{i},y_{i}\right)$, i∈{1,…,m}, the objective is to find the vector of parameters Θ that makes the best fit to the model defined in Equation (2). Likewise, let $r\left(\Theta \right)={\left(r_{1}\left(\Theta \right),r_{2}\left(\Theta \right),\ldots ,r_{m}\left(\Theta \right)\right)}^{T}$ be the vector that holds the fitting errors between the data and the model so that $r_{i}\left(\Theta \right)=y_{i}-g\left(x_{i},\Theta \right)$.

Accordingly, the objective function F(Θ) to be considered is the sum of the squares of r(Θ) using the Euclidean norm as a metric, as follows:

(3) $$\begin{array}{c} F\left(\Theta \right)={∥r(\Theta )∥}_{2}^{2}=\sum_{i=1}^{m}{\left(y_{i}-g\left(x_{i},\Theta \right)\right)}^{2}. \end{array}$$

Since finding the vector Θ that minimizes this objective function is equivalent to minimizing the fitting error, and for the sake of solution feasibility, the optimization problem can be established as $\min_{\Theta \in R^{n}}F\left(\Theta \right)$.

In this work, the widely-used the Gauss-Newton method is chosen to find an optimal solution [31]. Hence, Θ is obtained in an iterative search from an initialization value using the expression:

(4) $$\begin{array}{c} \Theta_{k+1}=\Theta_{k}-{\left[J_{r}{\left(\Theta_{k}\right)}^{T}J_{r}\left(\Theta_{k}\right)\right]}^{-1}J_{r}{\left(\Theta_{k}\right)}^{T}r\left(\Theta_{k}\right), \end{array}$$

where *k* represents the number of iterations, $J_{r}$ denotes the Jacobian matrix of the residual vector and ∇ is the gradient operator. $J_{r}\left(\Theta \right)$ is calculated using:

$$J_{r}\left(\Theta \right)\;=\;\;\left[\begin{array}{cccc} \frac{\partial r_{1}\left(\Theta \right)}{\partial \theta_{1}} & \frac{\partial r_{1}\left(\Theta \right)}{\partial \theta_{2}} & \cdots \; & \frac{\partial r_{1}\left(\Theta \right)}{\partial \theta_{n}}\\ \frac{\partial r_{2}\left(\Theta \right)}{\partial \theta_{1}} & \frac{\partial r_{2}\left(\Theta \right)}{\partial \theta_{2}} & \cdots & \frac{\partial r_{2}\left(\Theta \right)}{\partial \theta_{n}}\\ \vdots & \vdots & \ddots & \vdots \\ \frac{\partial r_{m}\left(\Theta \right)}{\partial \theta_{1}} & \frac{\partial r_{m}\left(\Theta \right)}{\partial \theta_{2}} & \cdots & \frac{\partial r_{m}\left(\Theta \right)}{\partial \theta_{n}} \end{array}\right]\;=\;\left[\begin{array}{c} \nabla r_{1}{\left(\Theta \right)}^{T}\\ \nabla r_{2}{\left(\Theta \right)}^{T}\\ \vdots \\ \nabla r_{m}{\left(\Theta \right)}^{T} \end{array}\right].$$

Many of the methods used in optimization are based on a trust-region-type approach, which results appropriate for approximation problems. In a trust-region algorithm, the approximate model is only reliable in a region close (being neighbor) to the current iteration [32]. Such a neighborhood can be represented as a ball in some norm, the radius $\Delta_{k}$ updates from one iteration to another, according to how accurately the model approximates the objective function on the trial point [33].

In addition, as trust-region methods are based on the classical Levenberg-Marquardt method for nonlinear equations using approximations of the Hessian matrix, they become efficient computationally. From (4), it can be inferred that the iterative method for a given initialization value is:

(5) $$\begin{array}{c} \Theta_{k+1}=\Theta_{k}-{\left[J_{r}{\left(\Theta_{k}\right)}^{T}J_{r}\left(\Theta_{k}\right)+\Delta_{k}I\right]}^{-1}J_{r}{\left(\Theta_{k}\right)}^{T}r\left(\Theta_{k}\right), \end{array}$$

where $\Delta_{k}$ is a positive scalar and I is the identity matrix of order *n*.

A more relevant approach to the Gaussian model is expressing the exponential function as a power, as seen in (6). For large *n*, a useful approximation can be obtained using:

(6) $$\begin{array}{c} e^{x}\approx {\left(1+\frac{x}{n}\right)}^{n}, \end{array}$$

which has a low implementation cost, as shown in (14). In this case, model (2) can be expressed as:

(7) $$\begin{array}{cc} f_{\mathrm{Gauss}}\left(x\right) & \approx {\tilde{f}}_{\mathrm{Gauss}}\left(x\right)\\ & =\sum_{i=1}^{N}A_{i}{\left[1+\frac{\left(-{\left(\left(x-\mu_{i}\right)/\sigma_{i}\right)}^{2}\right)}{n}\right]}^{n}. \end{array}$$

Algorithm 1 summarizes the steps to calculate the parameters of a non-linear model such as the Gaussian model. Finally, as the mathematical statements presented above are expressed in terms of the generic independent variable *x*, as well as the dependent variables $f_{\mathrm{Gauss}}\left(x\right)$, and ${\tilde{f}}_{\mathrm{Gauss}}\left(x\right)$, the correspondence of variables for CF purposes is mentioned below:

- The electric current *I* is *x*,
- while the voltage *v* can be either $f_{\mathrm{Gauss}}\left(I\right)$ or the approximation ${\tilde{f}}_{\mathrm{Gauss}}\left(I\right)$.

**Algorithm 1:** Unconstrained nonlinear optimization procedure. **Input:** Measured dataset ${\left\{(x_{i},y_{i})\right\}}_{i=1}^{m}$  1: Use the mathematical model defined by Equation (2)  2: Determine the specific objective function F(Θ) to be minimized through Equation (3)  3: Calculate the residual vector $r_{i}\left(\Theta \right)=y_{i}-g\left(x_{i},\Theta \right)$  4: Determine the Jacobian matrix $J_{r}\left(\Theta \right)$  5: Use a Non-linear Least Squares algorithm to estimate the optimal parameters as described in Equation (5) **Output:** The vector parameter Θ

## 4. Experimental Results

**Figure 2:**
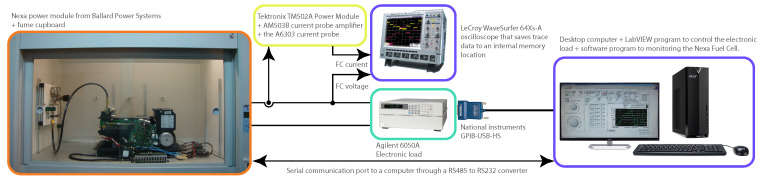
Experimental data adquisition configuration used for the Gaussian model training and validation.

The Nexa fuel cell is a fully integrated system that produces unregulated DC power, up to 1.2 kW, from a supply of hydrogen and air. The Nexa power module comes with LabVIEW software, which provides a graphical user interface to the operational status and performance of the Nexa module [34]. Figure 2 shows the experimental Nexa PEMFC data acquisition configuration used for training and validation of the Gaussian model.

The LeCroy WaveSurfer 64Xs-A oscilloscope has been used to achieve fast acquisition, long capture time, and data saving on its onboard hard drive. An oscilloscope is used to directly acquire and store the data corresponding to the fuel cell current and voltage signals. Thus, the maximum sampling limitation of the Nexa software is avoided, achieving sampling periods up to 20 μs. In addition, a virtual instrument is developed using LabVIEW, that generates the current profiles through the DC electronic load control using its GPIB communication port like a constant current load.

### 4.1. Training Models

**Figure 3:**
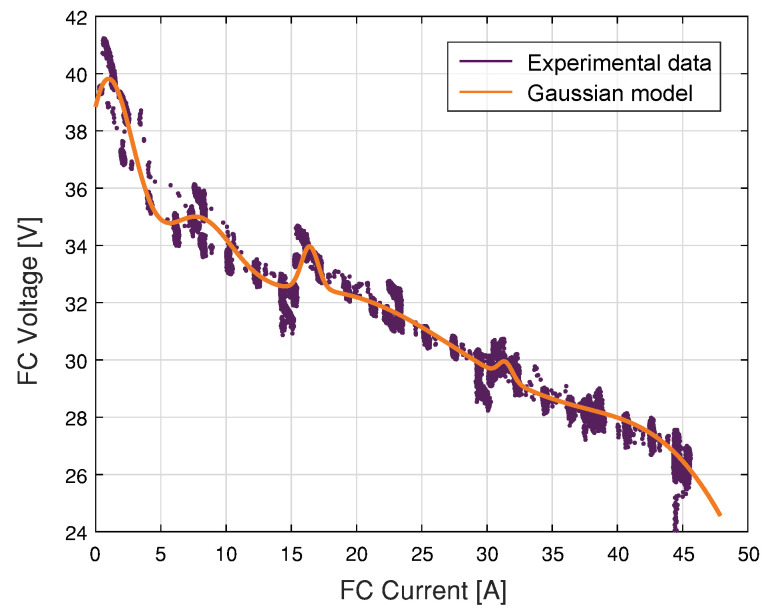
V-I characteristics of FC and Gaussian model.

**Figure 4:**
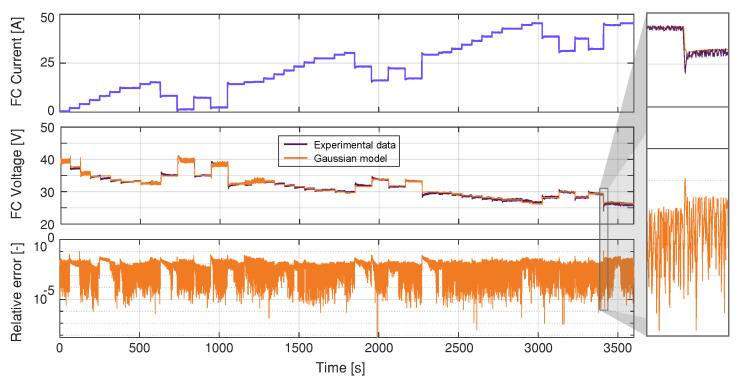
FC Gaussian model training.

This work proposes a Gaussian model to estimate the voltage in a different current region of the fuel cell. Specifically, the Nexa fuel cell case that has current training data between 0 A and 45 A. Figure 3 represents the voltage-current characteristics of the Gaussian model and the measured data. The Gaussian model responses agree with the real data from the fuel cell. In addition, a FC load current profile is generated, as shown in Figure 4, to reproduce the different operating points and transients to train the model in the entire operating current FC subdomains.

It can be observed that the load profile is provided in A (current) instead of A/cm${}^{2}$ (current density of active area). It is well-known that the current density allows easy comparison between different FC systems. However, the information about the Nexa active area is not provided by the manufacturer. Meanwhile, the literature reports different values such as 100 cm${}^{2}$ in [35], and 110 cm${}^{2}$ in [36], among others.

As for the Gaussian model parameters, as widely discussed in [37,38], the number of peaks *N* of Equation (2) may vary from 1 (a single Gaussian component) to a given maximum number $N_{\max}$. As a Taylor series approximation is subsequently applied, $1\leq N_{\max}\leq 8$ is an advisable search interval. In this work, a sub-optimal value of *N* is obtained experimentally as 6. Likewise, the value of *n* is swept in an adequate interval as is explained in Section 4.3. In such vein, the optimal values of the model coefficients of (2) are obtained following the trust-region Equation (5). Therefore, the appropriate mathematical Gaussian model for the characteristics of the data is described as a sum of six Gaussian peaks, as seen in model of Equation (8). Therefore, this approach requires 18 parameters to established.

The resulting Gaussian model is given by (8):

(8) $$\begin{array}{cc} f_{\mathrm{Gauss}}\left(I\right)= & \;12.43\cdot e^{\left(-{\left(\left(I-0.4927\right)/3.372\right)}^{2}\right)}\\ \; & +4.438\cdot e^{\left(-{\left(\left(I-7.067\right)/4.409\right)}^{2}\right)}\\ \; & +1.522\cdot e^{\left(-{\left(\left(I-16.4\right)/0.8977\right)}^{2}\right)}\\ \; & +32.39\cdot e^{\left(-{\left(\left(I-16.72\right)/36.84\right)}^{2}\right)}\\ \; & +0.5889\cdot e^{\left(-{\left(\left(I-31.42\right)/0.7715\right)}^{2}\right)}\\ \; & +8.769\cdot e^{\left(-{\left(\left(I-47.17\right)/12.34\right)}^{2}\right)}. \end{array}$$

It can observed that, as the current drawn from the FC increases, the FC voltage decreases; additionally, the simulated FC voltage closely follows the experimental FC voltage. The most significant deviation between the experimental voltage values and the ones estimated by the model happens at 3410.74 s, corresponding to a current step of 44.45 A. At this point, the voltage difference is 2.75 V, corresponding to a relative error of 11.46%. This point is observed in the zoomed view presented in Figure 4. The modeling results are quantified in Figure 4 using root mean square error (RMSE), defined as:

(9) $$RMSE=\sqrt{\frac{\sum_{t=1}^{n}{\left(y_{k}-{\hat{y}}_{k}\right)}^{2}}{n_{p}}},$$

where $\left(y_{k}-{\hat{y}}_{k}\right)$ is the error between the measured and the estimated FC output voltages, and $n_{p}$ is the number of steps of the discrete signal. The obtained results are considered satisfactory, given the fact that, for the proposed model, the RMSE is 0.27 V.

### 4.2. Validating Model

**Figure 5:**
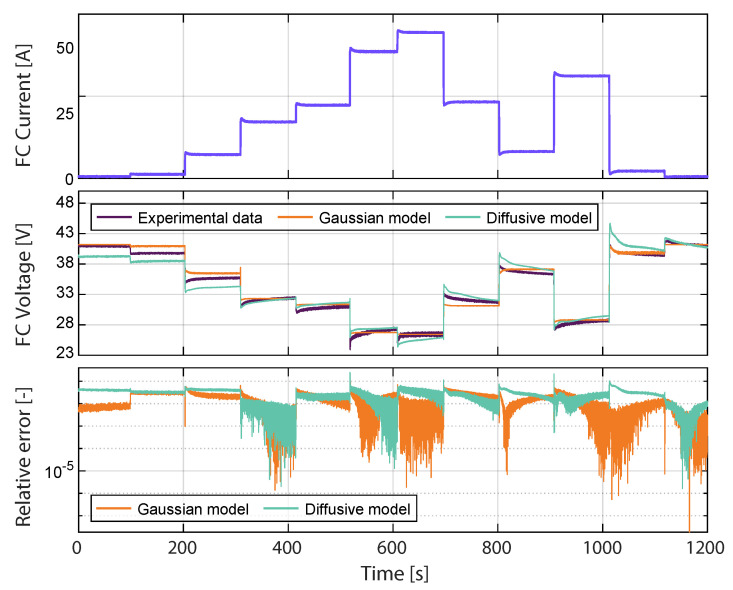
Validation of the FC Gaussian model.

**Figure 6:**
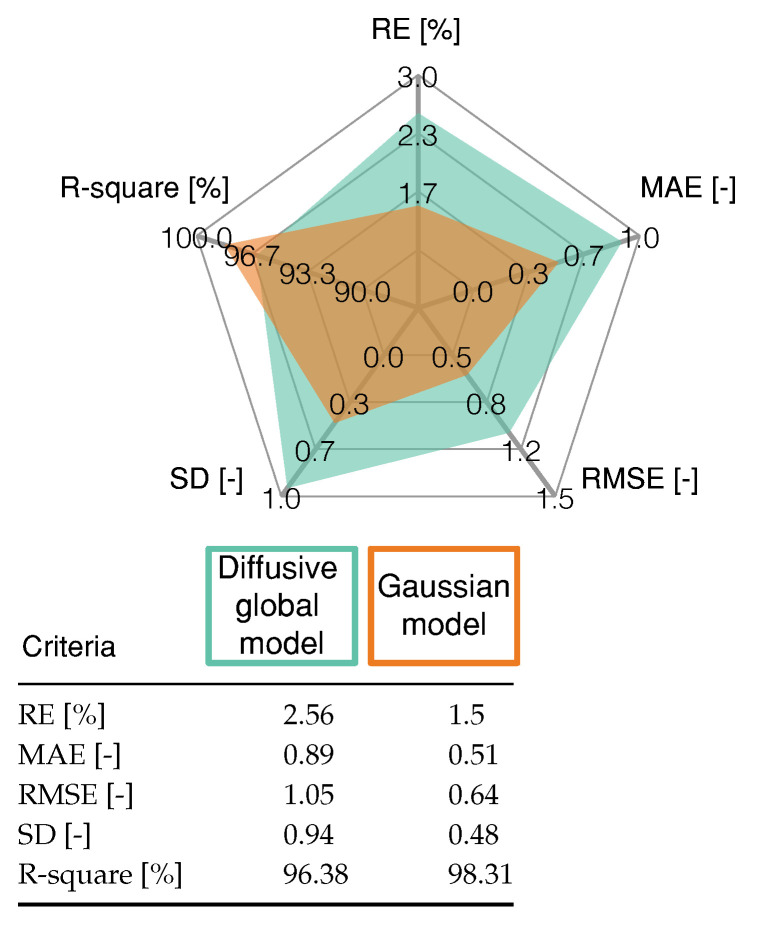
Statistical results of proposed Gaussian model and the Diffusive global model for the profile shown in Figure 5.

The validation of the proposed model is carried out by the test shown in Figure 5, which is different from the one used during training. This current profile is much more demanding than the one used for the validation stage because it has current step changes that are higher in magnitude. The proposed model is compared with the diffusive approach introduced and widely studied in [24]. The maximum deviation for both models happens at 518.14 s for 38.42 A. At this point, the difference between the Gaussian model voltage value and the experimental one is 2.86 V, with a relative error of 11.97%. For the diffusive model, the voltage difference is 4.87 V, with a relative error of 24.7%. Therefore, the Gaussian model fits the experimental data for validation better than the diffusive model, with an RMSE of 0.65 V for the Gaussian model and 1.05 V for the Diffusive model. Figure 6 shows the sensitivity of the FC models regarding different metrics. The mathematical expressions for R-square, relative error (RE), mean absolute error (MAE), and standard deviation (SD) are:

(10) $$\begin{array}{ccc} \mathrm{R-square} & = & \frac{n_{p}\sum_{t=1}^{n_{p}}y_{k}{\hat{y}}_{k}-\sum_{t=1}^{n_{p}}y_{k}\sum_{t=1}^{n_{p}}{\hat{y}}_{k}}{\sqrt{\left(n_{p}\sum_{t=1}^{n_{p}}y_{k}^{2}-{\left(\sum_{t=1}^{n_{p}}y_{k}\right)}^{2}\right)\left(n\sum_{t=1}^{n_{p}}{\hat{y}}_{k}^{2}-{\left(\sum_{t=1}^{n_{p}}{\hat{y}}_{k}\right)}^{2}\right)}}\times 100\%, \end{array}$$

(11) $$\mathrm{RE}=\frac{\sum_{t=1}^{n_{p}}\left(y_{k}-{\hat{y}}_{k}\right)}{{\hat{y}}_{kmean}}\times 100\%,$$

(12) $$\mathrm{MAE}=\frac{\sum_{t=1}^{n_{p}}\left(y_{k}-{\hat{y}}_{k}\right)}{n},$$

and

(13) $$\mathrm{SD}=\sqrt{\frac{\sum_{t=1}^{n_{p}}\left(y_{k}-{\hat{y}}_{k}\right)}{n_{p}-1}},$$

where $y_{k}$ represents the estimated voltage, ${\hat{y}}_{k}$ is the measured voltage, and ${\hat{y}}_{kmean}$ is the mean value of the measured voltage. The statistical analysis -presented in Figure 6 shows that the proposed Gaussian model has a low error and a high R-square value, compared with the Diffusive global model.

(14) $$\begin{array}{ccc} {\tilde{f}}_{\mathrm{Gauss}}\left(I\right) & = & 8.079\cdot {\left(1+\left(-{\left(\left(I-\left(-2.601\right)\right)/3.56\right)}^{2}/n\right)\right)}^{n}+1.572\cdot 10^{+11}\cdot {\left(1+\left(-{\left(\left(I-\left(-1154\right)\right)/238.8\right)}^{2}/n\right)\right)}^{n}\\ & & +1.28\cdot {\left(1+\left(-{\left(\left(I-22.7\right)/0.1055\right)}^{2}/n\right)\right)}^{n}+27.41\cdot {\left(1+\left(-{\left(\left(I-17.41\right)/95.78\right)}^{2}/n\right)\right)}^{n}. \end{array}$$

### 4.3. Comparison of Gaussian Model with the Parameter Identification by Means of Evolution Strategy

**Figure 7:**
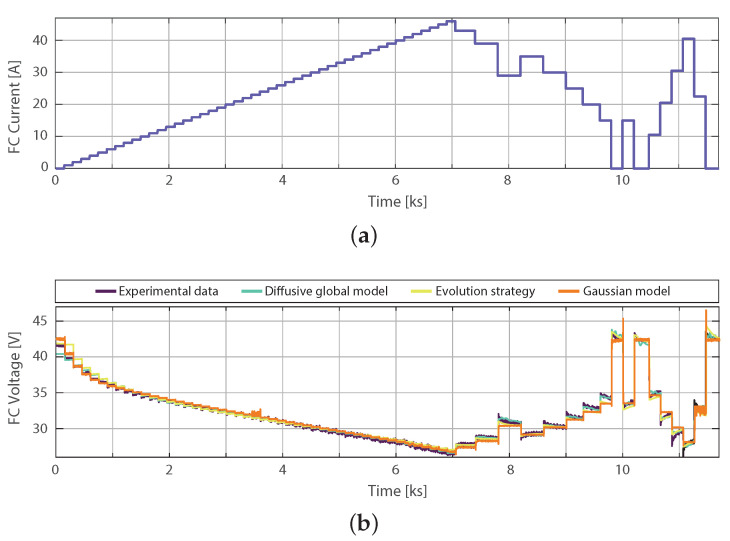
Experimental Nexa FC data used for training: (**a**) current load profile, (**b**) output voltage simulated with parameters estimated by means of the ES, the diffusive global model and Gaussian model.

An approach based on parameter identification of an equivalent circuit-based proton-exchange membrane fuel cell model is introduced in [8] using an ES. Training and validation data were sampled within a period of 200 ms in [8], which is 10 times higher than the sampling in the profile in Figure 4. Therefore, models are, again, both validated and trained, the current profile used for training the Gaussian model can be observed in Figure 7a, and the Diffusive Global model is introduced in [24]. The training model results are illustrated in Figure 7. This figure shows the experimental response of the fuel cell to the load current profile shown in Figure 7b. The RMSE of the Diffusive Global model is 0.3648 V, for the parameters adjusted by ES, 0.7961 V, and for the Gaussian approach it is 0.3567 V. Therefore, the diffusive approach and the Gaussian model have similar predictions of the output voltage. For its low implementation cost, the exponential function of the Gaussian model can be used as shown in the approximation (14) with n=265.

**Figure 8:**
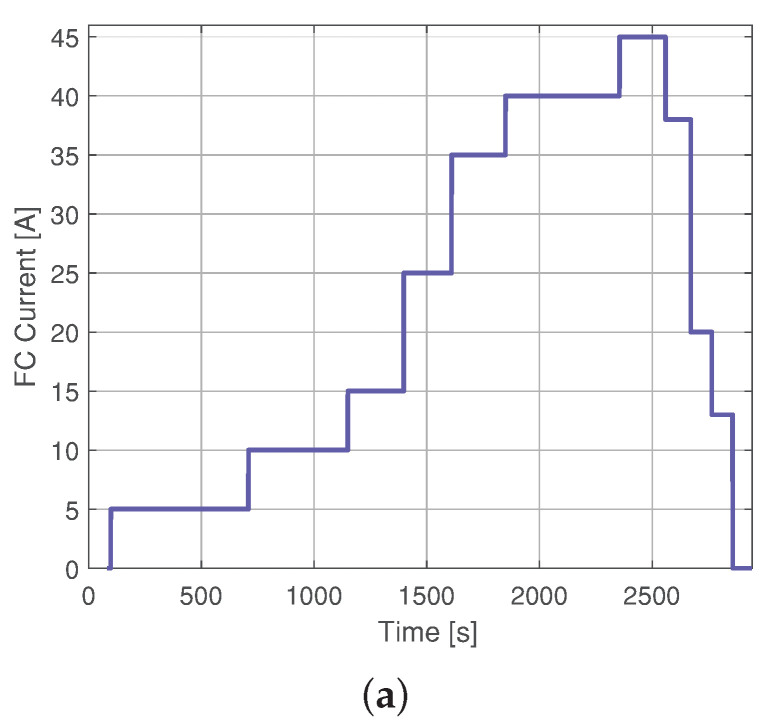

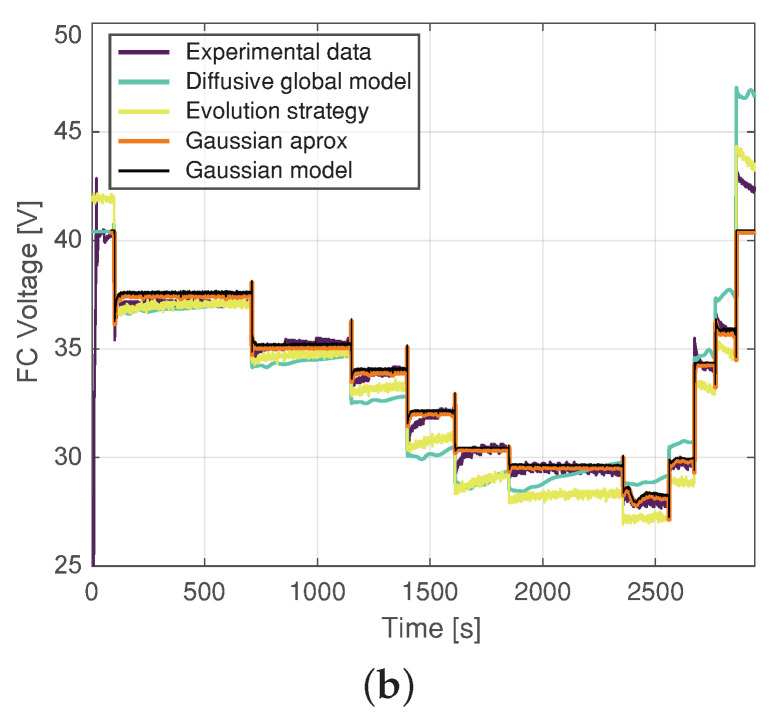
Experimental Nexa FC data used for validating: (**a**) current load profile and (**b**) output voltage simulated with parameters estimated by means of ES, the diffusive global model and the Gaussian model.

**Figure 9:**
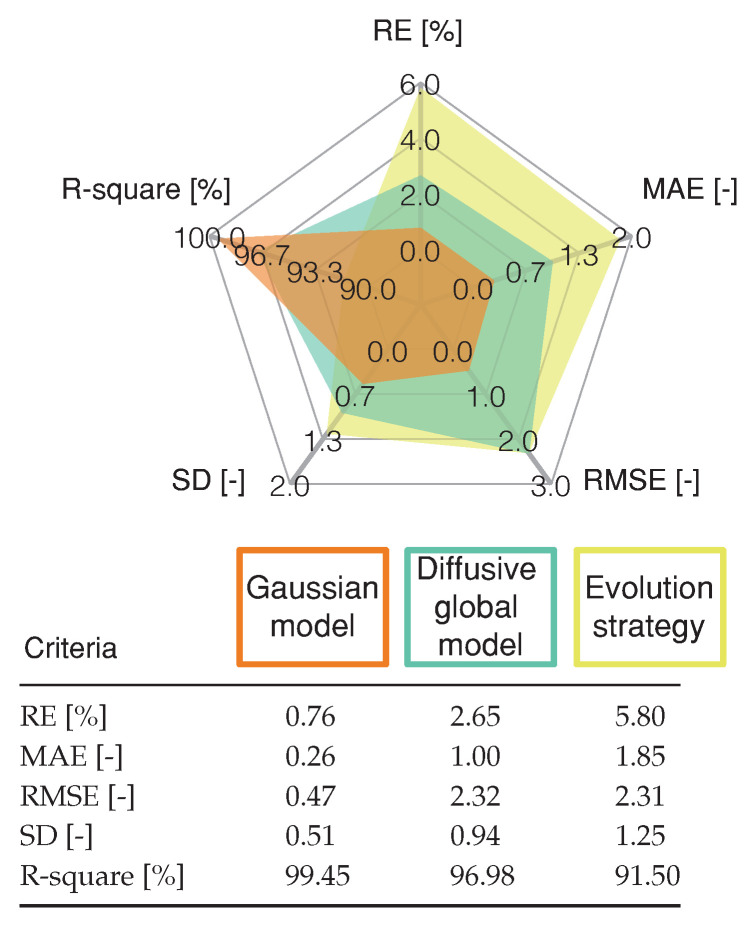
Statistical results of proposed Gaussian model, Diffusive global model and ES approach for the profile shown in Figure 8.

Finally, Figure 8 shows the validation of both approaches, where the RMSE of the diffusive global model and the parameters adjusted by ES were 2.3273 V and 2.3116 V, respectively. Meanwhile, the RMSE for the Gaussian approach is 0.47 V. The statistical results of the proposed Gaussian model are detailed in Figure 9. These results are compared with those from the Diffusive global model and those from the ES approach. The Gaussian model has the lowest error value, as it accurately represents the static and the dynamic current-voltage relation in a PEMFC.

## 5. Conclusions

This work develops a PEMFC model based on the Gaussian approach to estimate the FC voltage for the steady-state and dynamic responses. The results from the proposed model show similar behavior to those obtained on the experimental data with the Ballard Nexa 1.2 kW FC. Different training and validation profiles are developed to compare the proposed model with the Diffusive global model and the Evolution strategy based on numerical and analytical techniques. As a remarkable result, the Gaussian model reached superior performance, and its effectiveness was validated using statistical measures. In addition, an alternative model is also validated using an approximation to the exponential function that can be used in hardware emulators due to its lower-complexity implementation.

## Figures and Tables

**Table 1:** Fuel cell models comparison.

FC Model Strategy	Ref.	Static Model	V-I Dynamic Model	Variables Used to Evaluate the Model	Training Complexity	Implemen-Tation Cost	Tested with a Real FC
CHHO	[7]	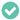	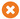	$T_{fc}$, $i_{fc}$, $P_{H_{2}}$, $P_{O_{2}}$, $R_{m}$	M	H	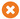
GOA	[25]	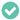	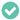	$T_{fc}$, $i_{fc}$, $P_{H_{2}}$, $P_{O_{2}}$, $R_{m}$	L	H	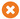
GWO	[16]	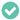	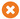	$T_{fc}$, $i_{fc}$, $P_{H_{2}}$, $P_{O_{2}}$, $R_{m}$	L	H	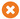
HGA	[18]	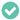	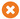	$T_{fc}$, $i_{fc}$, $P_{H_{2}}$, $P_{O_{2}}$, $R_{m}$	L	H	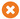
Electrical circuit	[26]	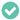	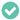	$T_{fc}$, $i_{fc}$, $P_{H_{2}}$, $P_{O_{2}}$, $R_{m}$	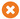	H	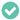
MAEO	[19]	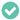	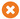	$T_{fc}$, $i_{fc}$, $P_{H_{2}}$, $P_{O_{2}}$, $R_{m}$	L	H	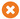
VSDE	[20]	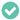	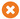	$T_{fc}$, $i_{fc}$, $P_{H_{2}}$, $P_{O_{2}}$, $R_{m}$	M	H	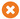
ASO	[21]	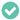	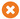	$T_{fc}$, $i_{fc}$, $P_{H_{2}}$, $P_{O_{2}}$, $R_{m}$	H	H	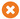
Electrical model	[6]	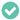	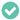	$T_{fc}$, $i_{fc}$, $P_{H_{2}}$, $P_{O_{2}}$, $R_{m}$	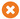	H	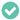
MPA-PO	[27]	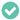	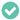	$T_{fc}$, $i_{fc}$, $P_{H_{2}}$, $P_{O_{2}}$, $R_{m}$	M	H	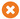
TS-KF	[28]	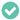	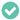	$T_{fc}$, $i_{fc}$	H	H	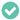
ARX-RLS	[10]	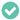	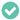	$T_{fc}$, $i_{fc}$, $P_{H_{2}}$, $P_{O_{2}}$, $R_{m}$	L	H	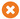
Bézier Curve	[22]	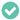	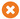	$i_{fc}$	M	H	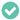
ES	[8]	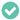	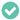	$T_{fc}$, $i_{fc}$, $P_{H_{2}}$, $P_{O_{2}}$, $R_{m}$	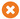	H	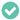
Diffusive model	[24]	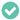	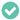	$i_{fc}$	H	M	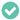
This work	[-]	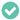	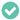	$i_{fc}$	M	L	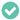

## Data Availability

Not applicable.

## Abbreviations

The following abbreviations are used in this manuscript:

ARX	Auto regressive eXogenous
ASO	Atom search optimization
CHHO	Chaotic Harris Hawks optimization.
ES	Evolution strategy.
FC	Fuel cell.
GOA	Grasshopper optimisation algorithm.
GWO	Grey wolf optimizer.
HGA	Hybrid genetic algorithm.
KF	Kalman filter.
MAE	Mean absolute error.
MAEO	Modified Artificial Ecosystem Optimization.
MPA	Marine Predators Algorithm.
PEMFC	Proton exchange membrane fuel cell.
PO	Political Optimizer.
RE	Relative error.
RLS	Recursive least square.
SD	Standard deviation.
VSDE	Vortex search differential evolution.
